# Whole exome sequencing reveals *heparan sulfate proteoglycan 2* (*HSPG2*) as a potential causative gene for kidney stone disease in a Thai family

**DOI:** 10.1007/s00240-024-01674-0

**Published:** 2024-12-16

**Authors:** Oranud Praditsap, Nawara Faiza Ahsan, Choochai Nettuwakul, Nunghathai Sawasdee, Suchai Sritippayawan, Pa-thai Yenchitsomanus, Nanyawan Rungroj

**Affiliations:** 1 Division of Molecular Medicine, Research Department, Faculty of Medicine Siriraj Hospital, Mahidol University, Bangkok, Thailand; 2 Immunology Graduate Program, Department of Immunology, Faculty of Medicine Siriraj Hospital, Mahidol University, Bangkok, Thailand; 3 Division of Nephrology, Department of Medicine, Faculty of Medicine Siriraj Hospital, Mahidol University, Bangkok, Thailand; 4 Siriraj Genomics, Office of the Dean, Faculty of Medicine Siriraj Hospital, Mahidol University, Bangkok, 10700 Thailand

**Keywords:** *Heparan sulfate proteoglycan 2*, *HSPG2*, Kidney stone disease, Whole exome sequencing

## Abstract

Kidney stone disease (KSD) is a prevalent and complex condition, with an incidence of 85 cases per 100,000 individuals in Thailand. Notably, over 40% of cases are concentrated in the northeastern region, indicating a potential genetic influence, which is supported by genetic mutations reported in several families by our research group. Despite this, the genetic basis of KSD remains largely unknown for many Thai families. This study aimed to identify the genetic mutation responsible for KSD in a specific Thai family, the UBRS131 family, which includes four affected individuals. Whole exome sequencing was performed, and variant filtering using the VarCards2 program identified 10 potentially causative mutations across 9 genes. These mutations were subjected to segregation analysis among family members and screened in 180 control and 179 case samples using real-time PCR-HRM or PCR-RFLP techniques. Prioritization of these variants using GeneDistiller identified the p.Asp775Glu mutation in the *heparan sulfate proteoglycan 2* (*HSPG2*) gene as the likely causative mutation for KSD in this family. The Asp775 residue is highly conserved across vertebrates, and structural analysis suggests that the Glu775 substitution may disrupt the formation of two crucial hydrogen bonds, potentially altering the mutant protein’s configuration. Immunohistochemistry confirmed the presence of perlecan (HSPG2 protein) in the proximal tubules in nephrons. These findings highlight the significant role of the *HSPG2* gene in familial KSD within this study family.

**Supplementary Information:**

The online version contains supplementary material available at 10.1007/s00240-024-01674-0.

## Introduction

Kidney stone disease (KSD) is a common multifactorial condition that affects approximately 10% of adults worldwide. Various factors, including environmental conditions, lifestyle choices, and genetic predispositions contribute to an increased risk of stone formation. While genetic predisposition has been well established in 35–65% of patients, and monogenic contributions are estimated in up to 30% of stone formers, the molecular genetics underlying KSD remains poorly understood [1, 2]. Investigating the genetic etiology of KSD is further complicated by the disease’s heterogeneity and its interaction with environmental factors. Nonetheless, genome-wide association studies (GWAS) and candidate gene analysis have identified common variants associated with KSD [3]. Additionally, whole exome sequencing (WES) has facilitated the identification of causative mutations in genes such as *SLC25A25*,* SLC34A1*,* and ADCY10* [4–6].

In Thailand, KSD is highly prevalent in the northeastern region, accounting for over 40% of the country’s annual KSD cases. A genetic influence, with a relative risk (λ_R_) of 3.18, has been reported among family members of KSD patients [7]. Previous research by our group has demonstrated associations between KSD and genetic variants in the *F2*, *PARQ6*, and *ITLN1* genes, as identified through candidate gene association studies and GWAS [8, 9]. More recently, we have identified causative variants for KSD in the *SCN10A* and *PBK* genes using a WES approach [10, 11]. Despite these advancements, the genetic etiology of KSD remains unresolved for many affected Thai families, where recurrent stone episodes, multiple affected members, and the high cost of invasive treatment procedures collectively constitute a significant socio-economic burden [3].

Continuing our efforts to elucidate the genetic etiology of KSD in Thai families, this study aimed to identify the genetic mutation responsible for KSD in a Thai family via WES. Through intensive prioritization analysis, we successfully identified a specific causative mutation in *HSPG2* gene for the index family. This gene plays roles in diverse cellular processes such as bone and cartilage formation, cell adhesion, inflammation and wound healing, cancer angiogenesis, cardiovascular development, and autophagy. Our study provided the first genetic evidence of *HSPG2* mutation in a Thai family with KSD and confirmed the heterozygous mutation of this gene as a cause of KSD.

## Materials and methods

### Ethics approval

The use of human kidney tissues, blood, and urine samples in this study was approved by the Human Research Ethics Committee of the Siriraj Institutional Review Board (SIRB), Faculty of Medicine Siriraj Hospital, Mahidol University, Bangkok, Thailand (COA no. Si 392/2012 and COA no. Si 133/2015). Written informed consent was obtained from all subjects prior to conducting the study. All methods were performed in accordance with the relevant guidelines and regulations.

## Subject recruitment and sample collection

In this study, 180 patients with KSD and their families were recruited from Sappasitthiprasong Hospital in Ubon Ratchathani province, Thailand. The diagnosis of KSD in all cases was confirmed using various methods including radiography of kidney-ureter-bladder (plain KUB), ultrasonography, surgical scar with medical record of kidney stone operation, and/or clinical history of kidney stone and associated symptoms (i.e., back and abdominal pain, hematuria, and stone passage). Since patient recruitment and sample collection were completed during fieldwork day, performing CT scans and 24 h urine collections was not possible. Exclusion criteria were applied to individuals with secondary conditions, namely - renal tubular acidosis, primary hyperparathyroidism, inflammatory bowel disease, Cushing’s disease, and hyperthyroidism. These secondary conditions were identified through a combination of clinical history, symptom assessment, acute acid loading tests, blood and urine biochemical tests, and electrolyte analyses.

In addition, 180 healthy controls were also recruited from the same geographical area. All control subjects underwent examination using the same protocol applied to KSD patients and their families to confirm the absence of KSD. Genomic DNA extracted from peripheral blood mononuclear cells of patients and control subjects, using the standard phenol-chloroform method, was employed for subsequent genetic analyses.

## Human kidney tissues and cell lines

Fresh frozen kidney tissues were obtained from patients without KSD, which were the remaining specimens from routine pathological examinations. These kidney tissues, along with HEK293, HEK293T, HK2, HepG2, A549, and NK92 cell lines were employed to investigate *HSPG2* mRNA and protein expression through reverse transcription and polymerase chain reaction (RT-PCR) and immunochemistry, respectively.

## Whole exome sequencing and analysis

Genomic DNA of all family members from an index family (UBRS131), consisting of four individuals affected with KSD and four unaffected individuals, were sent to Macrogen (Seoul, South Korea) for whole exome sequencing (WES) analysis. WES was performed using an Agilent Sure Select Target Enrichment Kit (Illumina, San Diego, California, USA), and sequenced with Illumina HiSeq 2000/2500 (Illumina, Inc., San Diego, California). The reads obtained from WES were mapped against UCSC hg19 (http://genome.ucsc.edu/) using the Burrows-Wheeler Aligner (BWA, http://bio-bwa.sourceforge.net/). The SAM/BAM files were processed to locate and remove duplicate molecules using Picard (http://broadinstitute.github.io/picard/). Variant discovery and genotyping were performed using Genome Analysis Toolkit (GATK), with emphasis on data quality, and raw variant discovery and annotation were carried out using SnpEff.

## Bioinformatics analysis

Variants identified through WES were rigorously filtered and prioritized to identify candidate variations of interest. Considering the autosomal dominant inheritance pattern, only heterozygous variants present in the affected members and absent in unaffected members were included. The variants obtained from WES results were compared against the R256 gene panel (https://panelapp.genomicsengland.co.uk/panels/149/) combined with the ClinVar database to evaluate the known variants associated with nephrocalcinosis and nephrolithiasis [12, 13]. The acquired variants were further filtered to encompass non-synonymous changes, stop-gain/loss mutations, short insertions or deletions (indels), and those with a minor allele frequency of less than 0.01. Inhouse variants combined with T-REx database (https://trex.nbt.or.th/) were used to exclude the common variants in our populations [14]. Potential deleterious effects on proteins were evaluated using VarCards2 (https://www.genemed.tech/varcards2/#/index/home) [15]. Variants predicted to be damaging by at least two out of three pathogenicity scores, including REVEL, SIFT, and PolyPhen, were further assessed for their functional impacts using GeneDistiller (https://www.genedistiller.org/), focusing expression and function in the kidneys and any known association with KSD. Briefly, gene variants that segregated exclusively in affected family members, had a frequency less than 0.01 in cases and controls, approximated the estimated LOD score, were reported in other KSD-affected families, and received high pathogenicity scores from VarCards2 and GeneDistiller were comprehensively analyzed to select the candidate gene. Variants were scored based on known genes or phenotypes to predict their potential link to KSD and urolithiasis. The selected candidate variant underwent multiple amino acid sequence alignment using the Clustal Omega, comparing sequences from human, chimpanzee, orangutans, gibbon, dog, cow, mouse, rat, chicken and zebrafish.

### Genotyping and genetic analysis

Validation, segregation analysis, and screening of the candidate variants identified via WES were conducted by genotyping in affected family members via PCR-high resolution melting (HRM) or PCR-RFLP methods. Primer pairs specific to the candidate variants were designed with Primer 3 v.0.4.0 software (https://bioinfo.ut.ee/primer3-0.4.0/), using the GRCh37 (https://grch37.ensembl.org/index.html) as a reference genome, and obtained from Macrogen, Seoul, South Korea (Table S1). Real-time PCR-HRM was performed in a 10 µl reaction containing 100 ng of DNA template, 1x reaction buffer, 0.5 µM of each primer, 0.2 mM dNTPs, 2.0 mM MgCl_2_, 1.25 units of DNA polymerase (HS Prime Taq DNA polymerase; GeNet Bio Inc., Daejeon, South Korea), and 1x Resolight dye (Roche Diagnostics). Amplification began with initial denaturation at 94℃ for 10 min, followed by 35–40 cycles of denaturation at 94℃ for 20 s, annealing at primer-specific Ta determined via conventional PCR for 20 s, and extension at 72℃ for 15 s. The amplicons were immediately subjected to HRM analysis, beginning with denaturation for heteroduplex formation at 95°C for 30 s, cooling at 40°C for 30 s, followed by a continuous temperatures increase 60°C to 95°C at a rate of 1ºC per second with continuous data acquisition. Post-melting, the Gene Scanning module of the LightCycler^®^ 480 Software release 1.5.0. (Roche Applied Science, USA) was employed for data management. The melting curve patterns of samples were analyzed through normalization, temperature shifting and difference plots to distinguish between homozygous wild-type and heterozygous mutant variants.

PCR-RFLP was used for segregation analysis and subsequent screening in cases and controls for variants not conclusively genotyped via the real-time PCR-HRM. Primer was designed with dCAPS Finder 2.0 (http://helix.wustl.edu/dcaps/) to introduce restriction enzyme recognition sites into the nucleotide sequence. PCR products (3 µl) were digested in a reaction containing 1x Cutsmart buffer, and 1 U *HpyCH4III* (New England BioLabs, Beverly, MA, USA) and incubated at 37°C overnight. Digested products (5 µl) were denatured at 95°C for 5 min and loaded into a 10% non-denaturing polyacrylamide gel. Electrophoresis was performed at 150 V for 2.5 h. DNA bands were visualized by silver staining and documented using a scanner.

## Reverse transcription and polymerase chain reaction

Total RNA was extracted from fresh frozen kidney tissues and cell lines by using Trizol reagent (Invitrogen, California, USA) and converted to first-strand cDNA by reverse transcription (RT) reaction using the SuperScript™ III First-Strand Synthesis System (Invitrogen, California, USA). This cDNA was used to evaluate gene expression by amplifying three exon-exon junctions (exons 6–7, 32–33, and 56–57), with *GAPDH* cDNA as an internal control (primer sequences in Table S2).

Polymerase chain reaction (PCR) was performed in a 10 µl PCR reaction containing 1x reaction buffer, 0.25 mM dNTPs, 0.50 µM of each primer, 0.05 U Taq DNA polymerase, and 2.0 µl cDNA template. Thermal cycling conditions consisted of the following: initial denaturation at 95°C for 10 min, followed by 35 cycles of 95°C for 20 s, 58℃ for 20 s, and 72℃ for 30 s, with a final extension at 72℃ for 7 min. Gene expression was visualized and analyzed using the G: BOX chemiluminescence imaging system (Syngene, Cambridge, UK).

## Immunohistochemistry

The expression of the HSPG2 protein (perlecan) in paraffin-embedded human kidney tissues was evaluated using immunohistochemistry (IHC). Tissue sections were deparaffinized, rehydrated, and underwent antigen retrieval with 0.05% trypsin in a 2.5 mM EDTA solution for 30 min at 37°C. Non-specific binding was blocked with 2% bovine serum albumin (BSA). The tissues were then incubated overnight at 4ºC with the following primary antibodies: mouse anti-human perlecan monoclonal antibody (clone 7B5, 13–4400; Thermo Fisher Scientific) at 1:25, rabbit anti-AQP1 at 1:200, rabbit anti-AQP2 at 1:50, and mouse anti-human V-ATPase monoclonal antibody (sc-55544; Santa Cruz Biotechnologies) at 1:25. A secondary antibody conjugated to horseradish peroxidase (HRP) at 1:250 (Dako, Japan) was applied for 3 h. Sections were developed using 3% 3,3’-diaminobenzidine (DAB), counterstaining with hematoxylin and eosin, dehydrated, and examined with Axio Star Plus Light Microscope (Carl Zeiss Microscopy, Jena, Germany).

### Protein structure modeling and *in silico* mutagenesis

The three-dimensional structure of the wild-type perlecan protein, consisting of 4,391 residues, was generated using AlphaFold (https://alphafold.ebi.ac.uk/) [16]. To evaluate the impact of the p.Asp775Glu (p.D775E) variant on protein structure, computational analysis was conducted with the same sequence and subsequently modeled using the SWISS-MODEL homology modeling server (https://swissmodel.expasy.org/). Alterations in the chemical bond formation resulting from amino acid variation were examined using PyMOL 1.7.5.0 (DeLano Scientific LLC, Palo Alto, California, USA).

## Results

### Subjects and clinical study

**Fig. 1:**
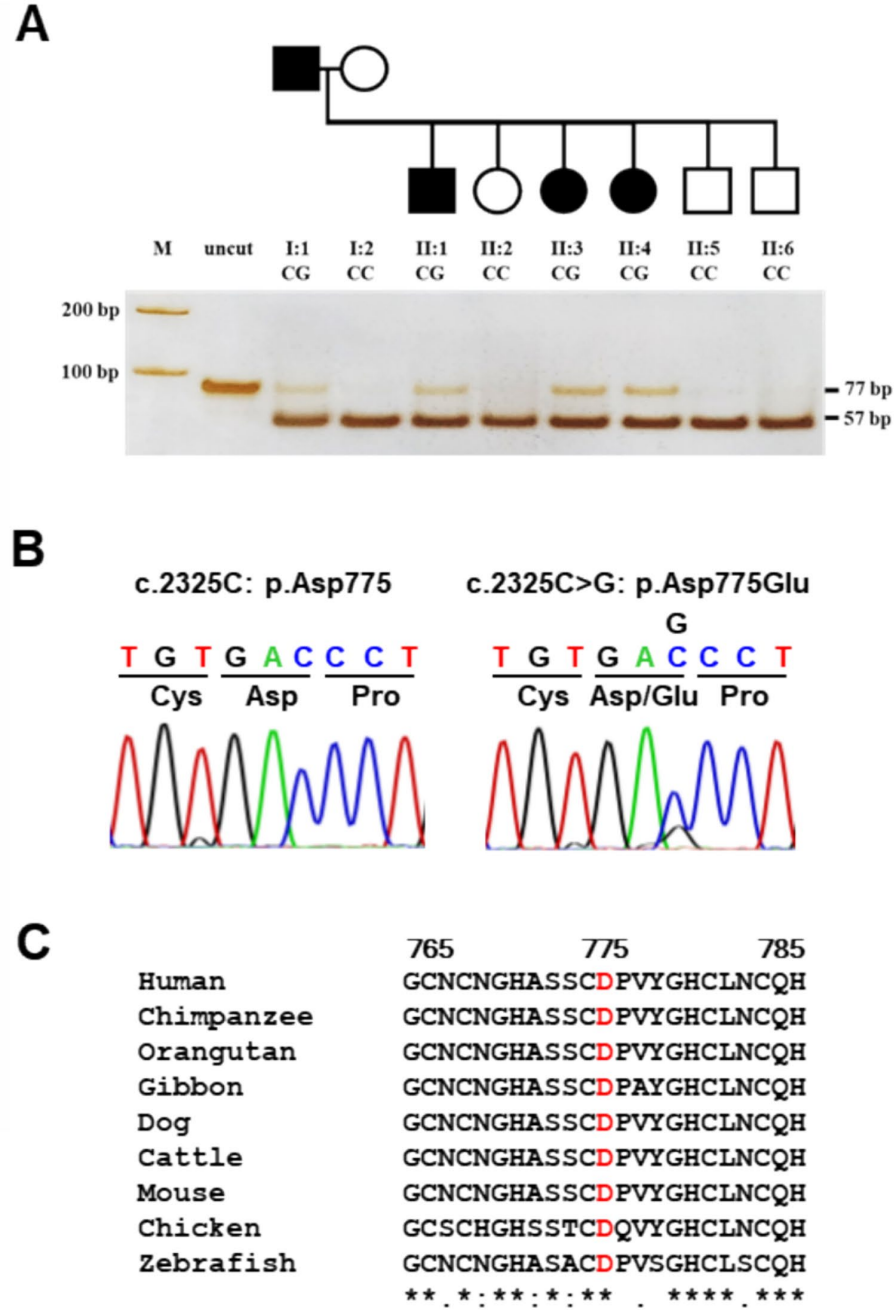
Pedigree of the UBRS131 family affected by KSD, segregation analysis of *HSPG2*, variant validation, and multiple amino-acid sequence alignment. **A** The pedigree of the UBRS131 family affected by KSD is presented alongside the segregation analysis of *HSPG2* c.2325C > G in eight family members using PCR-RFLP and polyacrylamide gel electrophoresis stained with silver. Black circles or squares represent affected individuals with KSD, and the genotypes of c.2325C > G is indicated below each symbol. **B** Sanger sequencing results confirm the present of the *HSPG2* c.2325C > G (p.Asp775Glu) variant in the affected patients. **C** Multiple amino acid sequence alignment of *HSPG2* from eight vertebrate species is shown for the region encompassing the p.Asp775Glu variation

The UBRS131 family, selected from a cohort of 180 affected families for this genetic study, showed no secondary causes of KSD based on comprehensive clinical history, symptom evaluations, acute acid loading tests, and serum electrolyte analyses. The family comprises eight members, with four (I:1, II:1, II:3, and II:4) across two generations diagnosed with KSD or urolithiasis. The pedigree (Fig. 1A) indicates an autosomal dominant inheritance pattern, potentially transmitted from the father (I:1) to three offspring (II:1, II:3, and II:4). Clinical and biological data (Table 1) show a pronounced phenotypic expression of KSD. KUB radiographies identified three stones in the kidney and ureter of I:1, two renal stones in II:1, and one stone in II:3 and II:4. KUB results from all affected members showed that all stones were opaque which represent calcium oxalate stones. Urinary pH levels were normal for all family members.

**Table 1:** Biological and clinical data collected from all members of the UBRS131 family

ID	Gender	Age	Age of onset	Symptoms	KUB results	Location of stone	Urine pH	No. of stones	Diagnosis
I:1	M	77	71	Dysuria, hematuria	Positive	Renal and ureter	5.6	3	Affected
I:2	F	68	–	Backpain	Negative	None	6.9	–	No stone
II:1	M	51	19	Dysuria, hematuria, backpain, pass stone, lower left abdominal pain	Positive	Renal	6.5	2	Affected
II:2	M	50	–	None reported	Negative	None	6.8	–	No stone
II:3	F	44	25	None reported	Positive	Ureter	6.0	1	Affected
II:4	F	43	43	Backpain	Positive	Renal	5.9	1	Affected
II:5	M	41	–	Backpain	Negative	None	5.5	–	No stone
II:6	M	38	–	None reported	Negative	None	5.2	–	No stone

### Whole exome sequencing analysis and variant filtering

To elucidate the genetic mutations underlying KSD in this family, WES was conducted on all family members. After ruling out the known variants from the R256 nephrolithiasis panel from Genomics England and ClinVar database, the WES analysis identified 57,207 variants across all individuals, with 419 variations exclusive to the affected members. These variants were further filtered to include insertions, deletions, stop/gain/loss, and splice site variants with a population frequency less than 0.01, narrowing the results to 25 mutations. Subsequent prioritization, based on the mutations’ impact and their pathogenicity scores, reduced the number to 10 variants across 9 genes (Fig. S1). These selected variants underwent further genetic analysis to identify the disease-causing gene. Table 2 details the WES findings and prediction scores obtained from VarCards2 for these variants of interest. The selected variants comprise five missense variants in *HSPG2*,* ARHGEF10*,* CNKSR1*, and *CDK11B*, and five splice region and intronic variants in *PER3*, *RERE*, *DOCK5*, *CCDC27*, and *OR8G5*. Notably, two missense variants, p.Asp775Glu and p.Val3123Met, were identified in the *HSPG2* gene.

**Table 2:** Summary of segregation analysis, screening, and prediction scores from VarCards2 and GeneDistiller for the variants of interest

Gene	Variant	Segregation Analysis (LOD score)	VarCards2						GeneDistiller Score	Heterozygous gentoype in controls (Number, frequency) *N* = 180	Heterozygous gentoype in cases (Number, frequency) *N* = 179	Potential Causative Variant
			REVEL		SIFT		PolyPhen					
			Score	*P*	Score	*P*	Score	*P*				
*HSPG2*	Missense variant c.2325C > G p.Asp775Glu	Yes (1.50)	0.478	N	0.171	T	0.997	PD	29.6	0	0	Yes
*ARHGEF10L*	Missense variant c.1568G > T p.Arg523Leu	Yes (1.50)	0.364	N	0.008	D	0.979	PD	8.3	0	0	Yes
*CNKSR1*	Missense variant c.422C > T p.Ser141Leu	Yes (1.50)	0.337	N	0.0	D	0.998	PD	8	1 (0.005)	0	Yes
*PER3*	Splice region variant & intron variant c.2188+ 5G > A	Yes (1.50)	NA	NA	NA	NA	NA	NA	8	0	0	Yes
*RERE*	Splice region variant & intron variant C.4339+ 5G > A	Yes (1.50)	NA	NA	NA	NA	NA	NA	17.41	1 (0.005)	1 (0.005)	Yes
*CDK11B*	Missense variant c.302A > G p.Lys101Arg	Yes (1.50)	0.146	N	0.0	D	0.99	D	2.6	5 (0.02)	3 (0.016)	Yes
*HSPG2*	Missense variant c.9367G > A p.Val3123Met	Yes (1.50)	0.448	N	0.03	D	0.999	PD	29.6	6 (0.033)	8 (0.044)	No
*DOCK5*	Splice region variant & intron variant c.1192+ 6_1192 + 36del	Yes (1.50)	NA	NA	NA	NA	NA	NA	7.3	10 (0.055)	9 (0.050)	No
*CCDC27*	Splice region variant & intron variant c.861+ 6 C > A	Yes (1.50)	NA	NA	NA	NA	NA	NA	0	7 (0.038)	2 (0.005)	No
*OR8G5*	Splice region variant & stop retained variant c.1040G > A p.Ter347Ter	No (1.50)	NA	NA	NA	NA	NA	NA	1	ND	ND	No

*NA* not applicable; *ND* not determined; *P* prediction; *N* neutral; *T* tolerated; *PD* probably damaging; *D* deleterious

### Segregation testing and validation of variations of interest in family members

The 10 variants of interest were genotyped in all of the family members using real-time PCR-HRM or PCR-RFLP methods. These genotyping results confirmed the findings from the WES analysis. All variants of interest were co-segregated in affected family members with a LOD score of 1.50 (Table 2; Fig. 1A), except for the p.Ter347Ter variant in the *OR8G5* gene, which was not detected by real-time PCR-HRM analysis or Sanger sequencing, suggesting a false positive result in the WES analysis. Further screening of these variants was conducted in 180 controls and 179 cases from the same geographical area. Genotyping of variants in controls allowed the identification of common polymorphisms with a frequency greater than 1%, thus prioritizing rare variants with potentially pathogenic outcomes. Screening results revealed heterozygous variants, in both control and case groups for *RERE*, *CDK11B*, *HSPG2* (p.Val3123Met), *DOCK5*, and *CCDC27*, indicating these variants were likely polymorphisms and less pathogenic. However, no heterozygous genotypes were found for p.Asp775Glu in *HSPG2*, p.Arg523Leu in *ARHGEF10L*, and c.2188+ 5G > A in *PER3* in either case or control groups. A summary of results from segregation testing, screening in cases and controls, and prediction programs is in Table 2.

**Fig. 2:**
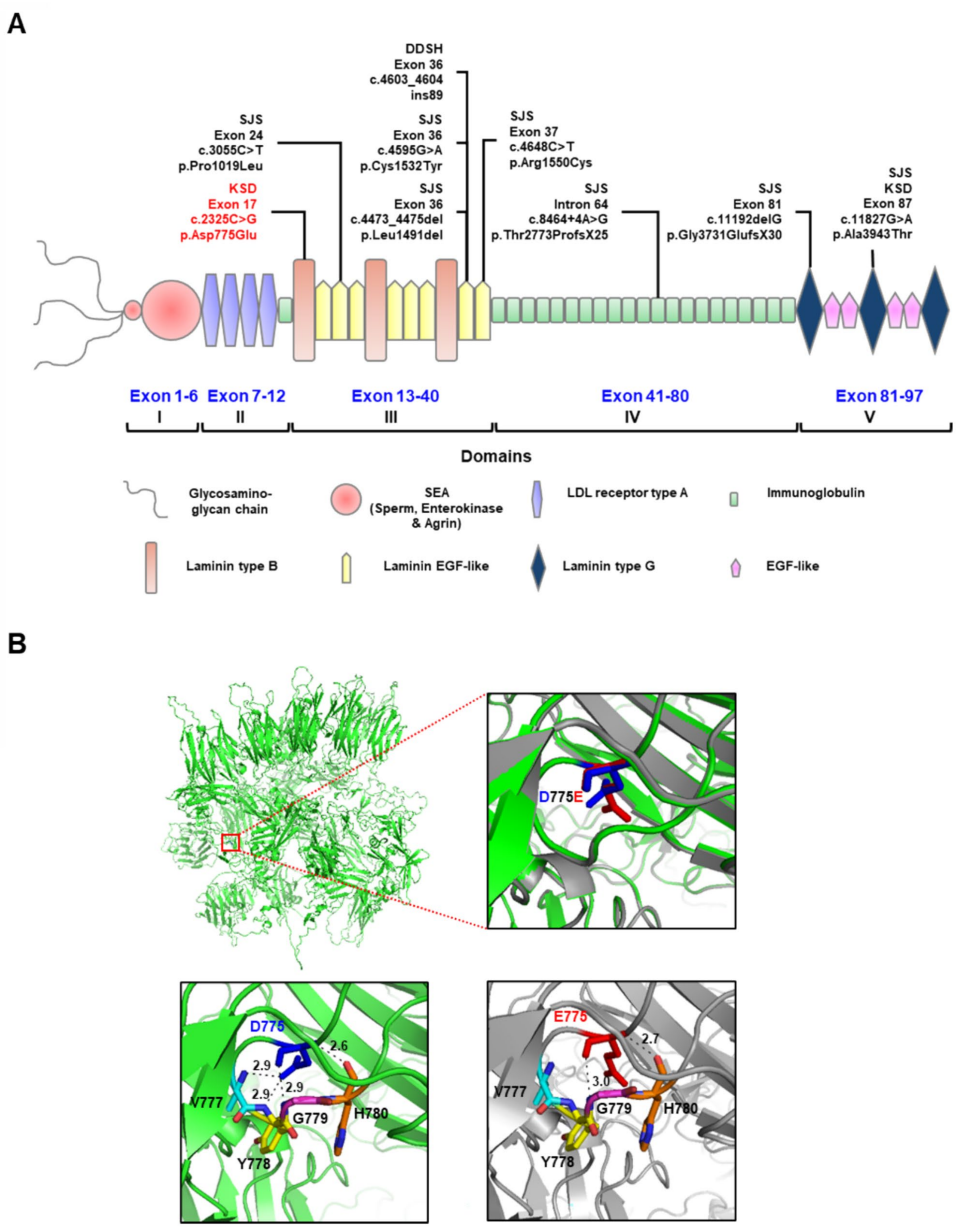
Schematic diagram and three-dimensional (3D) structure of perlecan. **A** The schematic diagram illustrates the structure of *HSPG2* (perlecan), highlighting the c.2325C > G (p.Asp775Glu) variation in exon 17 (red text) within domain III, along with other variations reported in previous studies. **B** Three-dimensional and superimposed structures of wild-type (green) and p.D775E (grey) perlecan. The square box indicates the protein region where the mutation is located. The structures of wild-type D775 (blue) and E775 variant (red), as predicted by SWISS-MODEL homology modeling server, are displayed along with their superimposed forms (blue and red). There are four putative H-bonds connecting to D775 in the wild-type structure (bottom-left, blue and green). In contrast, only two putative H-bonds connect to Glu775 (bottom-right, red and grey)

Although selection of the candidate gene responsible for KSD in this family was initially based on segregation analysis, screening in cases and controls, prediction scores from two web-based interfaces, and prior publications, the results from three variants (p.Asp775Glu in *HSPG2*, p.Arg523Leu in *ARHGEF10L*, and p.Ser141Leu in *CNKSR1*) appeared similar. To address this, further prioritization was conducted based on gene-phenotype associations, gene expression patterns, and protein-protein interactions using GeneDistiller [17]. This approach addressed the prioritization dilemma and facilitated the selection of the candidate gene, as demonstrated in previous studies [18–22]. By using additional criteria, the *HSPG2* gene carrying the variant c.2325C > G (p.Asp775Glu) emerged as the most promising candidate. This variant achieved the highest score of 29.6 in GeneDistiller and this *HSPG2* has been previously reported as an associated gene in KSD [23], indicating its potential functional role. Consequently, *HSPG2* was identified as the potential candidate gene for KSD in this family. This variant is located in exon 17 of domain III (Fig. 2A). Sanger sequencing validated this *HSPG2* variant, and Clustal Omega multiple amino acid sequence alignment revealed conservation of p.Asp775 (p.D755) across humans and analyzed vertebrates (Fig. 1B-C).

### Homology modeling and *in silico* mutagenesis

*HSPG2* encodes the basement membrane-specific perlecan protein, which is crucial for embryonic development, vasculogenesis, and maintaining homeostasis across basement membranes. The structure of this protein has been characterized [24]. We used a homology modeling approach to visualize the biophysical property changes in the protein structure resulting from the c.2325C > G, p.Asp775Glu (p.D775E) mutation. The three-dimensional structure of 4391 amino acid residues comprising the perlecan protein (Fig. 2B) was predicted using AlphaFold, yielding a predicted template modeling (pTM) scored 0.33, indicating a moderately accurate topology. The structure of mutated perlecan was generated through *in silico* mutagenesis and homology modeling using the SWISS-MODEL. Figure 2B illustrates the simulated structures of both wild-type and mutant perlecan. In the wild-type, residue Asp775 (D775) forms the hydrogen bonds (H-bonds) with Val777 (V777), Tyr778 (Y778), Gly779 (G779), and His780 (H780). In contrast, the mutant residue, Glu775 (E775) forms H-bonds only with Gly779 (G779) and His780 (H780), at different distances compared to the wild-type. Additionally, no extra intra- or inter-domain hydrogen interactions were observed in either the wild-type or mutant proteins.

### Investigation of mRNA and protein expression

**Fig. 3:**
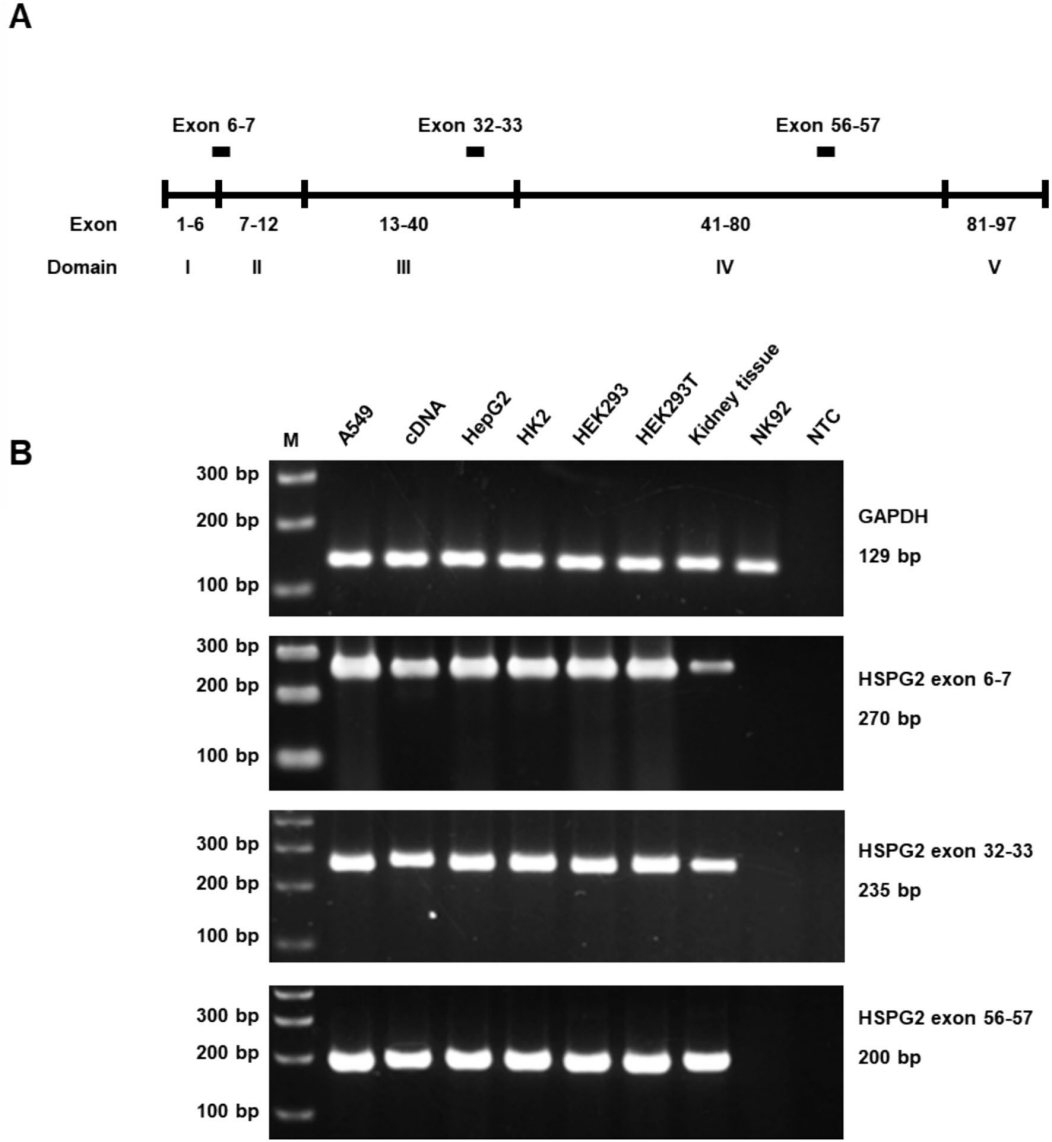
Expression of *HSPG2 *mRNA in kidney cell lines and human kidney tissues. **A** Primer map indicates the relative locations of primers used to amplify *HSPG2* mRNA. **B** *HSPG2* mRNA expression in A549 cells, kidney cDNA library, HepG2 cells, HK2 cells, HEK293 cells, HEK293T cells, and human kidney tissues detected by RT-PCR method. Three regions of *HSPG2* mRNA, spanning exons 6–7, 32–33, and 56–57, were analyzed. The mRNA of the housekeeping gene, *GAPDH*, served as an internal control. *NTC* no template control

The expression of *HSPG2* mRNA was examined in various human cell lines (A549, HepG2, HK2, HEK293, HEK293T, and NK92), kidney tissue, and kidney cDNA library using RT-PCR with three specific exon fragments. The A549 and NK92 cell lines served as positive and negative controls, respectively. GAPDH cDNA was amplified and used as an internal control, given its uniform expression across all cell lines (including the negative control NK92), and kidney tissues. Figure 3 shows that exon fragments 6–7, 32–33 (encoding domain III of protein), and exon 56–57 (encoding domain IV of protein) were expressed in the human kidney tissues, the kidney cDNA library, and all selected cell lines, except NK92.

**Fig. 4:**
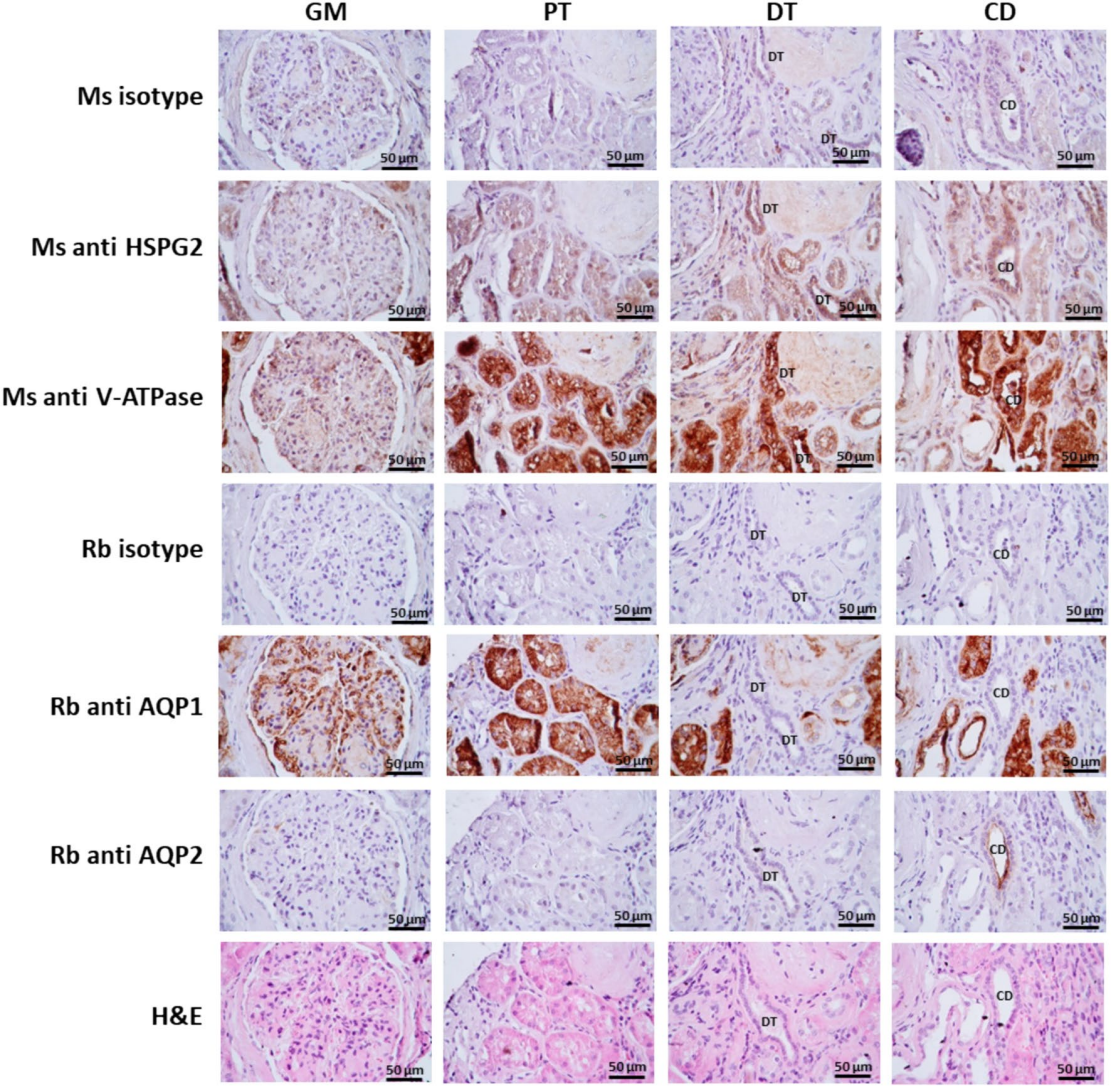
Expression of perlecan protein in human kidney tissues. Perlecan protein expression in the glomerulus and human kidney tubules was assessed by immunohistochemistry (IHC) using perlecan-specific antibody, with an isotype control antibody for comparison. AQP1 was used as a protein marker for the proximal tubule, V-ATPase as a protein marker for the distal tubule and collecting duct, and AQP2 as a protein marker for the collecting duct. The original magnification was 40x. The abbreviations are as follows: *Ms* mouse; *Rb* rabbit; *GM* glomerulus; *PT* proximal tubule; *DT* distal tubule; *CD* collecting duct; *H&E* hematoxylin and eosin staining. Scale bar: 50 μm

The expression of perlecan protein in human kidney tissues was also assessed by immunohistochemistry (IHC) assays. The results demonstrate prominent perlecan expression in the glomerulus and tubules (Fig. 4), with the highest expression observed in the proximal tubules. The distal tubules and collecting ducts exhibited slightly lower levels of expression.

## Discussion

The etiology of KSD is heterogeneous and remains unknown in many northeastern Thai families. In previous studies, our group has determined genetic variants associated with modified KSD risk in affected families from this region. To further elucidate the genetic basis of KSD in Thai families, we employed a WES approach. WES results identified 10 variants in 9 genes, with the SNV c.2325C > G in the *HSPG2* gene (NM_001291860.1) emerging as a potential causative variant in a Thai family (Table 2). This variant in exon 17 results in an amino acid substitution, p.Asp775Glu (rs149114054), which co-segregated with KSD in the affected index family (Figs. 1A-B and 2A). Although this variant was found in only the GO-ESP database (allele count 1/13006; allele frequency 0.00008), it was not detected in our cases or control group from the endemic area. Bioinformatic predictions prioritized the *HSPG2* variant due to its deleterious impact. Another variant, p.Val3123Met (rs530638947) in *HSPG2* gene, was detected in cis with the former. It was reported in the genomAD database (allele count 8/140282; allele frequency 0.000057) and was also found in our population, both controls and cases with a relatively high frequency (0.033 and 0.044), suggesting it is a polymorphism (Table 2).

Multiple amino acid sequence alignments revealed that the amino acid p.Asp775 (p.D775) is conserved across vertebrate species (Fig. 1C), underscoring its evolutionary significance for protein structure. A closer examination of the chemical structure shows that the wild-type aspartate (Asp; D) is replaced by the larger glutamate (Glu; E), both of which are negatively charged amino acids. Compared to Asp, Glu has a longer side-chain and higher molecular mass. Consequently, while the mutant protein may not change in charge or polarity, it exhibits a missing intra-chain H-bond within domain III (Fig. 2B). According to the *in silico* model, Asp775 is crucial for forming of H-bonds with nearby residues Val777, Tyr778, Gly779 and His780, whereas Glu775 cannot form H-bonds with Val777 and Tyr778. This substitution (p.Asp775Glu) likely alters H-bond formation patterns and distances, affecting protein stability and properties. Additionally, RNA expression analysis confirmed the expression of this gene in several human kidney cell lines (HK2, HEK293, and HEK293T) and the endothelial cell line (HepG2) (Fig. 3). Protein expression analysis demonstrated its presence along the nephron (Fig. 4). The robust expression of *HSPG2* mRNA and protein in the human kidney suggests a significant role of this gene in kidney function.

The *HSPG2* gene encodes the large multidomain proteoglycan perlecan, which is prominently found in all basement membranes, and the extracellular matrix (ECM) of skeletal and connective tissues. Perlecan is essential for numerous physiological processes, including embryonic development of chondrocytes, cardiovascular and neural tissues, vasculogenesis, and the maintenance of homeostasis across basement membranes. Additionally, perlecan promotes cell growth, differentiation, attachment, and adhesion, while modulation of physiological processes such as angiogenesis, tumorigenesis, apoptosis, and autophagy [25]. Previous studies have shown that homozygous mutations in the *HSPG2* gene lead to pathological outcomes in two rare genetic disorders affecting the musculoskeletal system: Schwartz-Jampel syndrome (SJS) and dyssegmental dysplasia, Silverman-Handmaker type (DDSH) [26]. SJS results from partial loss-of-function mutations in the *HSPG2* gene, leading to myotonia, chondroplasia, and neuromuscular deficits. In contrast, functionally null mutations cause DDSH, characterized by more severe skeletal and cephalic defects [25–27]. Additionally, mutations in this gene have been implicated in the pathogenesis of prostate cancer, Alzheimer’s disease, and coronary artery disease [28–30]. Although the mechanisms underlying these associations have not yet been elucidated, the lethal outcomes of null mutations in this gene, highlight its critical importance in human health. It is plausible that distinct amino acid changes, combined with interactions with other proteins in specific cell types, contribute to the disease phenotypes associated with these mutations.

Besides, variants in the *HSPG2* gene have also been associated with various skeletal and renal disorders, including scoliosis, diabetic nephropathy (DN), Balkan endemic nephropathy (BEN), and KSD. In the context of KSD, researchers investigated a specific SNP (rs3767140) located in intron 6 of the *HSPG2* gene among 143 individuals prone to CaOx stone formation and 158 healthy controls from the same region without any history or radiological signs of stone disease. This SNP was found to significantly elevate the risk of urolithiasis in a recessive model within the Turkish population [23]. Moreover, recent findings by Chatterjee et al. identified a heterozygous mutation at c.4489T > A (p.Phe1497Ile, rs138460117) in an 11 years old patient from West Bengal, India, who presented with early onset nephrolithiasis but no other clinical symptoms [31]. This mutation, located in domain III of perlecan, has been documented in the ClinVar database under accession numbers RCV000365542.3.

The variant in our KSD-affected family is located within domain III of perlecan, showing structural similarities to laminin B and laminin EGF proteins (Fig. 2A) [32]. Previous studies have documented a missense variant (c.3263T > C) in domain III that leads to SJS by disrupting perlecan secretion into the ECM [33]. Additionally, several other mutations in domain III have been observed in SJS cases, although their specific roles in SJS pathogenesis remain unclear. While variants in domain III associated with SJS typically do not correlate with renal defects, similar variants in other domains have broader effects beyond the musculoskeletal systems. For instance, the SJS-related variant c.11827G > A; p.Ala3943Thr in domain V is thought to enhance calcium deposition by altering hydrogen bonding, leading to renal manifestations such as juvenile nephrolithiasis and osteopenia in individuals with SJS [25].

It is well-documented that genetic renal disorders often manifest with musculoskeletal symptoms across various conditions. Mutations in the *CLC-5* gene responsible for Dent disease (DD) are known to cause skeletal abnormalities alongside renal symptoms like KSD and low molecular weight proteinuria [34]. Similarly, autosomal recessive disorders such as hereditary hypophosphatemic rickets with hypercalciuria (HHRH) are associated with musculoskeletal complications including bone pain, rickets, and limb deformities [35]. The precise mechanisms linking skeletal manifestations to renal diseases remain incompletely understood, although they likely involve mineral transport, calcium homeostasis, and vitamin D metabolism. Given the crucial role of the *HSPG2* gene in skeletal tissue development and the pathogenesis of skeletal disorders, investigating both SJS-associated and non-SJS variants in domain III holds promise for elucidating the mechanisms underlying stone formation. Nonetheless, the absence of skeletal deformities or related conditions in index patients from our KSD-affected family suggests an alternative pathogenic mechanism underlies their disease.

The severity of neuromuscular conditions such as SJS and DDSH depends on the mutation’s location within the *HSPG2* gene and the extent of the preserved protein core. This core influences the production and secretion of functional protein into various extracellular matrices (ECMs) [25]. Similar to SJS and DDSH, the heterozygous variant c.2325C > G identified in this Thai index family may reduce the production or secretion of perlecan. Prior research has demonstrated that the loss of perlecan is linked to the disruption of normal compartmental structures in bone and cartilage [36], with patients suffering from SJS exhibiting a predisposition to bone and cartilage loss [37–39]. Bone diseases are frequently associated with KSD since bone serves as the largest reservoir of calcium in the body. Consequently, the loss of perlecan may elevate urinary calcium excretion, thereby correlating with stone formation.

Heparan sulfate is a urinary macromolecule that modulates nucleation, growth, aggregation, and retention of crystals in the kidneys. Functionally, *HSPG* has been identified as an inhibitor for calcium oxalate crystallization, creating a charge barrier that impedes the attachment of calcium oxalate monohydrate (COM) crystal and prevents the progression of urothelial damage [40]. However, the mutant variant identified in our KSD family belongs to the same negatively charge amino acid group as the wild-type. This suggests it is unlikely to simply alter the charge involving in crystal formation. It is plausible that the mutant protein affects the structural conformation, potentially resulting in functional abnormalities or instability that causes kidney stone formation.

In addition to structure defects, perlecan plays a crucial role in various signaling pathways. Domain III of perlecan is vital for interactions with several ligands, including platelet-derived growth factor (PDGF), fibroblast growth factor (FGF) 7, and FGF18, which are essential for their functions. Notably, FGF7 has been implicated in embryonic development of kidneys [41, 42]. Injecting mouse models of nephrolithiasis with FGF7 resulted in urothelium proliferation and enhanced retention of calcium stones in the kidneys [43]. Furthermore, FGF7 is one of the phosphatonins, like FGF23, that regulate phosphate homeostasis. Interestingly, FGF7 can bind to both domain III and domain V, where mutation in the latter has previously been reported to cause early-onset nephrolithiasis in patients with SJS [25].

Although the precise mechanisms by which this protein contributes to KSD are not yet fully understood, this study provides genetic evidence implicating it in the pathogenesis of KSD within the index family, marking the first such report in adults. Future research should aim to validate the impact of this variant by examining protein expression level, protein stability, and the signaling pathways related to mineral homeostasis and kidney stone formation.

## Conclusions

The integration of WES analysis and variant filtering, coupled with segregation analysis in family members, screening in control and case cohorts, and functional prediction studies, collectively identified *HSPG2* as the potential causative gene for KSD in the affected UBRS131 family.

## Supplementary Information

Below is the link to the electronic supplementary material.

Supplementary material 1 (PDF 170 KB)

## Data Availability

No datasets were generated or analysed during the current study.
