# Supplementary material for: Whole exome sequencing reveals heparan sulfate proteoglycan 2 (HSPG2) as a potential causative gene for kidney stone disease in a Thai family
Source: Urolithiasis. 2024 Dec 16;53(1):7. doi: 10.1007/s00240-024-01674-0 (PMC11649748; doi:10.1007/s00240-024-01674-0)
Supplement: Supplementary file 1 — Supplementary material 1 (PDF 170 KB) [file 240_2024_1674_MOESM1_ESM.pdf]

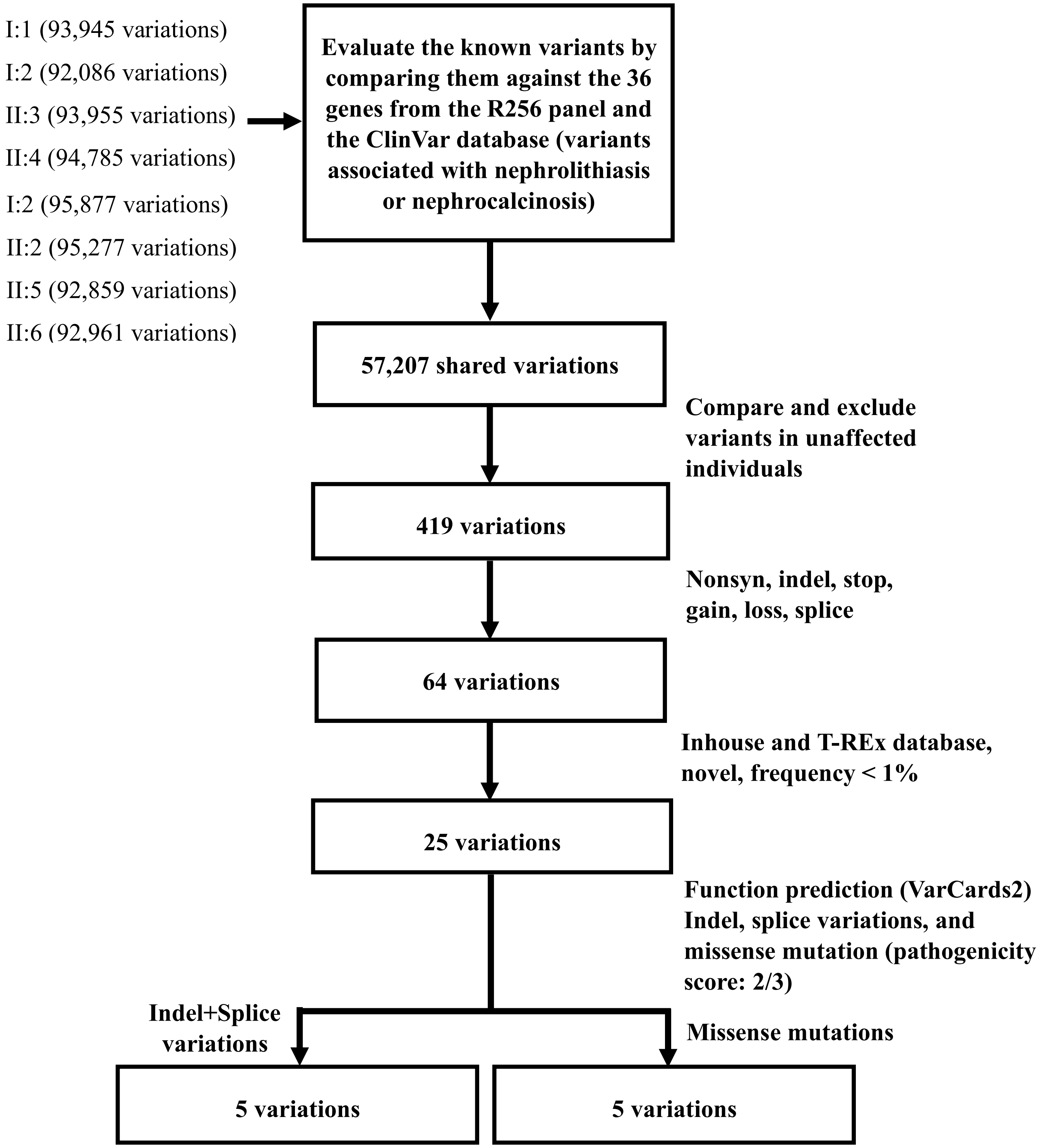

I:1 (93,945 variations)
I:2 (92,086 variations)
II:3 (93,955 variations)
II:4 (94,785 variations)
I:2 (95,877 variations)
II:2 (95,277 variations)
II:5 (92,859 variations)
II:6 (92,961 variations)
Evaluate the known variants by comparing them against the 36 genes from the R256 panel and the ClinVar database (variants associated with nephrolithiasis or nephrocalcinosis)
57,207 shared variations
Compare and exclude variants in unaffected individuals
419 variations
Nonsyn, indel, stop, gain, loss, splice
64 variations
Inhouse and T-REx database, novel, frequency < 1%
25 variations
Function prediction (VarCards2) Indel, splice variations, and missense mutation (pathogenicity score: 2/3)
Indel+Splice variations
Missense mutations
5 variations
5 variations

Figure S1

Table S1. PCR primers for genotyping 10 genetic variations in UBRS131 family members, 180 unrelated patients, and 180 normal control subjects.

Gene	Variation	Primer Sequence (5'→3')	Length (bp)	Ta (°C)	Product Size (bp)
ARHGEF10L	p.Arg523Leu	TGAGAAGCTGAACGAGCAGA	20	60	105
		GCCCAGCTCACCTTGTTG	18		
CNKSR1	p.Ser141Leu	TCCCCTGCCAGGTACCTCTTC	21	60	120
		AAGCCTGGCCCTTGGTTCTC	20		
CDK11B	p.Lys101Arg	AAGATGATTCTTTGGCCATCA	21	60	147
		TGTCAAAGAAAGTAAATGCTTCT	21		
HSPG2	p.Asp775Glu	CTACCTGGGCACCTGCTCT	23	62	88
		GGCACTACTCACCAGGCAGT	19		
HSPG2	p.Val3123Met	CTGCCTGTGTGTCAGTGGTT	20	66	192
		ATATGTCCGCTGCTCCAACT	20		
CCDC27	NM_152492.3:c.861 +6C>A	ATGCAGCTGAAATGCCTTCT	21	60	150
		AGGGGCCTGTGTGTCACTA	20		
PER3	NM_016831.1:c.2164 +5G>A	GCAGGAGCAAAGCTAAATA	19	53	150
		CTCCATCAGTTTCTTACAAGT	19		
RERE	NM_012102.4:c.4339 +5G>A	AGGACCCCCTCCACCAAG	21	60	137
		CAGGGATGAGGGATCTGTTC	18		
DOCK5	NM_024940.8:c.1192 +7_1192+37del	TGAATCACAAAGGGCAAGGT	20	60	144
		GGATGGTCCCCAGCAGAG	18		
OR8G5	p.Ter347Ter	CAGCCTGAGGAATAAAGATGTC	22	53	168
		GAAAGAATGTTGACAGATGCAC	22		

Table S2. PCR primers for analysis of HSPG2 expression.

Primer Name	Nucleotide Sequence	Number of nucleotides	Ta (°C)	Product Size (bp)
HSPG2 ex6-7	AGGGACATGTCTGATGAGC	18	58	270
	TCCTCCTGTCCGTCGCAG	18		
HSPG2 ex32-33	GAACCCACAGCGAAACAG	18	58	235
	CACATCGGGGTCAGAGAGTG	20		
HSPG2 ex56-57	GACCCTGGACCTGAACTG	18	60	200
	TGACAAGGACTGAGGCTT	18		
